# Receipt of Medications for Chronic Disease During the First 2 Years of the COVID-19 Pandemic Among Enrollees in Fee-for-Service Medicare

**DOI:** 10.1001/jamanetworkopen.2023.13919

**Published:** 2023-05-17

**Authors:** Nancy E. Morden, Weiping Zhou, Ziad Obermeyer, Jonathan Skinner

**Affiliations:** 1 The Dartmouth Institute for Health Policy and Clinical Practice, Geisel School of Medicine at Dartmouth, Lebanon, New Hampshire; 2 UnitedHealthcare, Minnetonka, Minnesota; 3 School of Public Health, University of California, Berkeley

## Abstract

**Importance:**

During the first 2 years of the COVID-19 pandemic, inpatient and ambulatory care declined dramatically. Little is known about prescription drug receipt during this period, particularly for populations with chronic illness and with high risk of adverse COVID-19 outcomes and decreased access to care.

**Objective:**

To investigate whether receipt of medications was maintained during the first 2 years of the COVID-19 pandemic among older people with chronic diseases, particularly Asian, Black, and Hispanic populations and people with dementia, who faced pandemic-related care disruptions.

**Design, Setting, and Participants:**

This cohort study used a 100% sample of US Medicare fee-for-service administrative data from 2019 to 2021 for community-dwelling beneficiaries aged 65 years or older. Population-based prescription fill rates were compared for 2020 and 2021 vs 2019. Data were analyzed from July 2022 to March 2023.

**Exposure:**

The COVID-19 pandemic.

**Main Outcomes and Measures:**

Age- and sex-adjusted monthly prescription fill rates were calculated for 5 groups of medications commonly prescribed for chronic disease : angiotensin-converting enzyme inhibitors and angiotensin receptor blockers, 3-hydroxy-3-methylglutaryl coenzyme A (HMG CoA) reductase inhibitors (statins), oral diabetes medications, asthma and chronic obstructive pulmonary disease medications, and antidepressants. Measurements were stratified by race and ethnicity group and dementia diagnosis. Secondary analyses measured changes in the proportion of prescriptions dispensed as a 90-day or greater supply.

**Results:**

Overall, the mean monthly cohort included 18 113 000 beneficiaries (mean [SD] age, 74.5 [7.4] years; 10 520 000 females [58.1%]; 587 000 Asian [3.2%], 1 069 000 Black [5.9%], 905 000 Hispanic [5.0%], and 14 929 000 White [82.4%]); 1 970 000 individuals (10.9%) were diagnosed with dementia. Across 5 drug classifications, mean fill rates increased by 2.07% (95% CI, 2.01% to 2.12%) in 2020 and decreased by 2.61% (95% CI, −2.67% to −2.56%) in 2021 compared with 2019. Fill rates decreased by less than the mean overall decrease for Black enrollees (−1.42%; 95% CI, −1.64% to −1.20%) and Asian enrollees (−1.05%; 95% CI, −1.36% to −0.77%) and people diagnosed with dementia (−0.38%; 95% CI, −0.54% to −0.23%). The proportion of fills dispensed as 90-day or greater supplies increased during the pandemic for all groups, with an increase per 100 fills of 3.98 fills (95% CI, 3.94 to 4.03 fills) overall.

**Conclusions and Relevance:**

This study found that, in contrast to in-person health services, receipt of medications for chronic conditions was relatively stable in the first 2 years of the COVID-19 pandemic overall, across racial and ethnic groups, and for community-dwelling patients with dementia. This finding of stability may hold lessons for other outpatient services during the next pandemic.

## Key Points

**Question:**

Were the COVID-19 pandemic and its associated health care disruptions associated with a decrease in prescription drug receipt among patients with chronic illness, particularly in populations with high risk of adverse COVID-19 outcomes and decreased access to care?

**Findings:**

In this cohort study of 18 113 000 beneficiaries of fee-for-service Medicare, mean prescription fill rates decreased by 2.61% in 2021 compared with 2019. Declines did not differ across Asian, Black, Hispanic, and White subpopulations or for people diagnosed with dementia.

**Meaning:**

These findings suggest that rates of medication receipt for chronic conditions were surprisingly resilient among older US adults during the COVID-19 pandemic.

## Teaser

This cohort study examines changes in prescription fill rates of 5 groups of medications for chronic diseases during the first 2 years of the COVID-19 pandemic among enrollees in fee-for-service Medicare.

## Introduction

During the first 2 years of the COVID-19 pandemic, inpatient, emergency department and ambulatory service use declined dramatically, by more than 50% in some settings and time frames,^1,2,3^ but less is known about prescription drug receipt during this period.^4^ Prescription drugs constitute a key component of effective management of chronic conditions, day-to-day disease control, and prevention or slowing of disease progression over time. Many important chronic illnesses in the US older population (eg, diabetes, lung disease, cardiovascular disease, and depression) are treated effectively with regularly prescribed drugs; cessation of such treatments may be associated with near-term adverse health outcomes.^5,6,7,8^ Traditionally in the US, prescriptions for medications to manage these conditions are written and renewed by clinicians during regularly scheduled face-to-face visits, including wellness and chronic condition–monitoring visits. The traditional association between face-to-face visits and prescription receipt raises the question of how the pandemic’s disruption of in-person health care may have been associated with patient receipt of these maintenance medications. We explored this question through population-level measurement of prescription drug receipt before and during the pandemic.

To investigate an association between the pandemic’s disruption of health care and pharmacotherapy for chronic conditions, we used Medicare fee-for-service administrative data from 2019 to 2021 for community-dwelling beneficiaries aged 65 years or older. We measured monthly population-level prescription fill rates for 5 groups of medications commonly prescribed for chronic illness. Our analysis compared prepandemic (2019), early pandemic (2020), and later pandemic (2021) fill rates.

Rates of prescription fills at the national level may obscure sharp declines for underserved patient groups. For this reason, we considered prescription fill rates separately by race and ethnicity. We hypothesized that higher rates of COVID-19 and shortfalls in access to care among Black and Hispanic enrollees may have been associated with reduced fill rates in these populations.^9,10^ Additionally, we separately considered enrollees diagnosed with Alzheimer disease and related dementias (ADRD), who were at increased risk of excess mortality during the COVID-19 pandemic,^11^ while care and quality of life for these patients were compromised owing to lockdowns and other restrictions.^10,12^

In secondary analyses, we measured proportions of fills before and during the pandemic occurring as 90-day or greater supplies. This was done given that larger supplies may be associated with decreased risk of patients running out of medications when lockdowns or fear could limit pharmacy access.

## Methods

This cohort study was approved by the National Bureau of Economic Research (NBER) Institutional Review Board (IRB) and conducted on the Centers for Medicare and Medicaid Services (CMS) Virtual Research Data Center, with a CMS data use agreement approval for Medicare 2019 to 2021 administrative data. The NBER IRB deemed this study exempt from requiring notification of Medicare enrollees because the research used a publicly available database subject to Health Insurance Portability and Accountability Act of 1996 (HIPAA) restrictions (45 CFR §46.104[d][4]), and it involved no analysis for which written consent was required. This study followed the Strengthening the Reporting of Observational Studies in Epidemiology (STROBE) reporting guideline.

We used 100% Medicare claims data for 2019 to 2021 for beneficiaries enrolled in fee-for-service Part D plans with concurrent Part A and B coverage who were aged 65 years or older at the beginning of each month and lived in the US. Because residents of nursing home and long-term care facilities receive prescriptions through their facility (and thus have less risk of supply disruptions associated with public health measures or fear), we restricted our sample to enrollees who were not institutionalized, defined as those with no evidence of nursing home residence during the month. Nursing home use was based on *Current Procedural Terminology* (*CPT*) codes for place of service, outpatient file *CPT* codes, or any skilled nursing facility code (eAppendix 1 in Supplement 1). Prescription fill rates were calculated monthly for people who were alive, so measure denominators changed month to month. We assumed that the pandemic was not associated with substantial changes in the true prevalence of underlying chronic diseases themselves, allowing us to interpret changes in prescription fill rates relative to baseline as changes in clinician prescribing or patient fill behavior rather than changes in the prevalence of disease.

We created 6 cohorts from the overall sample. The first was the entire noninstitutionalized population of US beneficiaries of fee-for-service Medicare aged 65 years and older residing in the US with Parts A, B, and D enrollment. The second through fifth cohorts include beneficiaries with mutually exclusive race or ethnic identity categories: Asian or Pacific Islander, Black or African American, Hispanic, and non-Hispanic White (White); these were self-reported classifications augmented using Research Triangle Institute imputations included in the Master Beneficiary Summary File.^13^ Because of small samples sizes, we were unable to study other groups (American Indian, Alaska Native, unknown, and other race and ethnicity), which we combined as an other category; these groups were excluded from rate calculations; see eAppendix 2 in Supplement 1 for monthly enrollment rates overall and by subgroup. The sixth cohort included people with a diagnosis of ADRD appearing on a claim from 2019 to 2021 (see eAppendix 3 in Supplement 1 for diagnosis codes).

### Measures and Covariates

The primary outcome measure was the 30-day age- and sex-adjusted prescription fill rate. This was created by using First Databank^14^ data to identify National Drug Code lists for products in the drug groups of interest: angiotensin-converting enzyme inhibitors and angiotensin receptor blockers (ACEs and ARBs), 3-hydroxy-3-methylglutaryl coenzyme A (HMG CoA) reductase inhibitors (statins), oral diabetes medications, asthma and chronic obstructive pulmonary disease (COPD) medications, and antidepressants (eAppendix 4 in Supplement 1).^14^ We used the NDC list to identify fill events within each drug group in the Part D Event file. For our primary measure, 30-day fill rates per 1000 beneficiaries, we standardized fill events to 30-day supplies by dividing the fill event days-supply variable value by 30.^15^ We then normalized the fill rate to 30-day months to adjust for differences in fill rates arising from the different number of days in each month (28-31 days). Finally, we calculated fill rates adjusted by age category (5-year age intervals and ages ≥85 years) and sex using indirect standardization.^16^ A secondary outcome measure was the fraction of total drug supplies filled through prescriptions specifying 90-day or greater supplies. Age and sex covariates were obtained from the Master Beneficiary Summary File.

### Statistical Analysis

For our summary measures of prescription drug use, we compared 12-month mean fill rates in 2021 with those in 2019, reporting the percentage change. We also considered monthly fill rates from January 2019 to December 2021 for graphical presentations. We further considered as a summary index measure the unweighted mean of all 5 prescription drug fill rates in 2021 compared with 2019 and in 2020 compared with 2019; see eAppendix 5 in Supplement 1 for statistical details. Statistical analysis was performed using SAS statistical software version 9.4 (SAS Institute) and Stata statistical software version 17.0 (StataCorp). The a priori significance level was *P* ≤ .05, with a 2-sided significance test. Data were analyzed from July 2022 to March 2023.

## Results

The mean monthly number of beneficiaries for the entire sample meeting inclusion criteria in 2019 to 2021 was 18 113 000 beneficiaries (mean [SD] age, 74.5 [7.4] years; 10 520 000 females [58.1%]; 587 000 Asian [3.2%], 1 069 000 Black [5.9%], 905 000 Hispanic [5.0%], and 14 929000 White [82.4%]; 623 000 with other race or ethnicity [3.4%] were not further analyzed). In the full sample, there were 1 970 000 people diagnosed with ADRD (10.9%) (Table 1). While the overall number of people in the fee-for-service cohort was largely unchanged from January 2019 to December 2021, there was a decrease from 1 151 794 to 965 299 Black enrollees (5.6% annually) and from 939 690 to 862 624 Hispanic enrollees (2.8% annually), which was largely associated with more rapid growth in Medicare Advantage enrollment among these groups.^17^ Given that the ADRD group included anyone with an ADRD diagnosis during 2019 to 2021, there was a decrease from 2 476 379 individuals in January 2019 to 1 364 437 individuals in December 2021 (an annual decline of 15.4%) in this group owing to mortality (eAppendix 2 in Supplement 1).

**Table 1.** Characteristics of Study Groups

Characteristic	Enrollees, No.^a^					
	Overall	Asian	Black	Hispanic	White	ADRD^b^
Monthly enrollees, mean	18 113 000	587 000	1 069 000	905 000	14 929 000	1 970 000
Age, mean (SD), y	74.53 (7.36)	75.28 (7.74)	74.07 (7.47)	74.52 (7.57)	74.65 (7.36)	81.27 (8.22)
Sex, fraction (SD)						
Female	0.581 (0.493)	0.592 (0.491)	0.631 (0.483)	0.595 (0.491)	0.582 (0.493)	0.630 (0.483)
Male	0.419 (0.493)	0.408 (0.491)	0.369 (0.483)	0.405 (0.491)	0.418 (0.493)	0.370 (0.483)

Abbreviation: ADRD, Alzheimer disease and related dementias.

^a^ For cohort inclusion, beneficiaries had to be enrolled in fee-for-service Medicare Parts A, B, and D for at least 1 month after turning age 65 years for each year considered. Beneficiaries with claims evidence of institutional (nursing home) residence were excluded. The 623 000 beneficiaries with other race or ethnicity, included in the overall count, were not included in further analysis.

^b^ Members of this group had an ADRD diagnosis appear on at least 1 claim in 2019 to 2021.

In 2019, before the pandemic, fill rates per 1000 Medicare enrollees ranged from 101.4 fills (95% CI, 101.3-101.6 fills) for diabetes drugs to 461.5 fills (95% CI, 461.3-461.7 fills) for statins, and the mean fill rate across drug groups was 262.0 fills (95% CI, 261.8-262.2 fills) (Table 2). Overall, mean fill rates per 1000 enrollees for the cohorts of interest in 2019 were lowest for Black enrollees (239.8 fills; 95% CI, 239.0-240.5 fills) and highest for people diagnosed with ADRD (298.6 fills; 95% CI, 298.0-299.1 fills).

**Table 2.** Effective Fill Rates of Medications for Chronic Disease

Medication type	Fill rate, No./1000 population^a^					
	Overall	Asian	Black	Hispanic	White	ADRD^b^
ACE and ARB						
2019	380.08	387.37	380.91	403.19	378.72	365.11
2021	372.07	385.30	367.29	390.38	371.28	339.67
Difference, 2019 vs 2021, % (95% CI)^c^	−2.11 (−2.16 to −2.06)	−0.54 (−0.82 to −0.25)	−3.58 (−3.79 to −3.37)	−3.18 (−3.40 to −2.95)	−1.97 (−2.02 to 1.91)	−6.97 (−7.12 to −6.81)
Statins						
2019	461.51	501.65	426.72	433.97	464.32	458.58
2021	455.19	501.48	427.70	423.43	457.03	454.09
Difference, 2019 vs 2021, % (95% CI)^c^	−1.37 (−1.42 to −1.32)	−0.03 (−0.29 to 0.22)	0.23 (0.03 to 0.44)	−2.43 (−2.65 to −2.21)	−1.57 (−1.62 to −1.52)	−0.98 (−1.12 to −0.83)
Diabetes drugs						
2019	101.44	198.81	133.91	174.60	90.19	111.30
2021	100.20	200.80	135.07	174.05	89.28	107.83
Difference, 2019 vs 2021, % (95% CI)^c^	−1.22 (−1.29 to −1.16)	1.00 (0.68 to 1.33)	0.86 (0.61 to 1.12)	−0.31 (−0.58 to −0.05)	−1.01 (−1.08 to −0.94)	−3.12 (−3.31 to −2.93)
Asthma drugs						
2019	115.63	116.05	121.97	111.52	116.04	130.43
2021	110.01	106.52	122.41	106.32	110.21	127.56
Difference, 2019 vs 2021, % (95% C)^c^	−4.86 (−4.92 to −4.80)	−8.21 (−8.54 to −7.58)	0.36 (0.11 to 0.61)	−4.66 (−4.93 to −4.30)	−5.03 (−5.10 to −4.96)	−2.20 (−2.39 to −2.02)
Antidepressants						
2019	251.31	105.30	135.31	192.82	271.69	427.39
2021	238.26	101.30	129.34	183.12	256.06	457.93
Difference, 2019 vs 2021, % (95% CI)^c^	−5.19 (−5.25 to −5.14)	−3.80 (−4.14 to −3.47)	−4.41 (−4.66 to −4.17)	−5.03 (−5.29 to −4.78)	−5.75 (−5.81 to −5.69)	7.14 (6.99 to 7.30)
Mean of 5 groups^d^						
2019	261.99	261.84	239.76	263.22	264.19	298.56
2020	267.39	269.58	246.61	266.85	266.31	306.53
2021	255.15	259.08	236.36	255.46	256.77	297.41
Difference, % (95% CI)						
2019 vs 2020	2.07 (2.01 to 2.12)	1.71 (1.42 to 1.99)	2.86 (2.64 to 3.07)	1.38 (1.14 to 1.61)	2.04 (1.98 to 2.10)	2.67 (2.52 to 2.82)
2019 vs 2021	−2.61 (−2.67 to −2.56)	−1.05 (−1.36 to −0.77)	−1.42 (−1.64 to −1.20)	−2.95 (−3.18 to −2.72)	−2.81 (−2.87 to −2.75)	−0.38 (−0.54 to −0.23)

Abbreviations: ACE, angiotensin-converting enzyme inhibitor; ADRD, Alzheimer disease and related dementias; ARB, angiotensin receptor blocker.

^a^ Rates are given for age- and sex-adjusted 30-day-equivalent fill rates per 1000 population by year. For cohort inclusion, beneficiaries had to be enrolled in fee-for-service Medicare Parts A, B, and D for at least 1 month after turning age 65 years for each year considered. Beneficiaries with claims evidence of institutional (nursing home) residence were excluded.

^b^ Members of this group had an ADRD diagnosis appear on at least 1 claim in 2019 to 2021.

^c^ The percentage difference is the change in rates divided by the initial use rate in 2019 and so is expressed as a percentage.

^d^ For the mean of all 5 groups, the percentage difference is the change in the mean index for 2020 or 2021 vs 2019 relative to the initial use rate in 2019.

**Figure 1.**
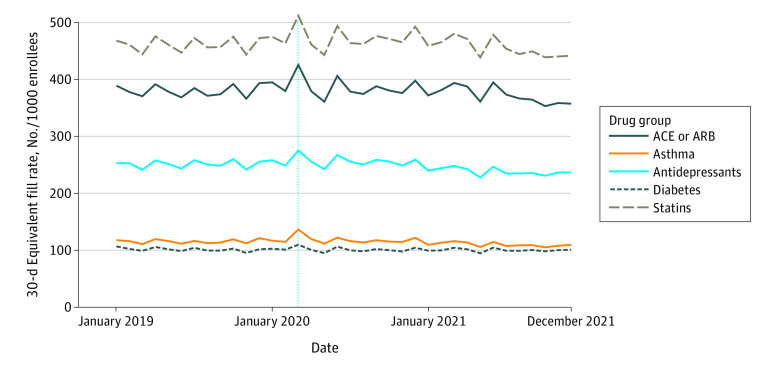
Fill Rates for 5 Groups of Medications Prescribed for Chronic Illness This figure displays monthly 30-day supply fill rates for 5 effective prescription drug groups during January 2019 to December 2021 for the entire cohort of enrollees in fee-for-service Medicare Parts A, B, and D. All rates are age and sex adjusted and per 1000 enrollees. The asthma medication group includes medications for asthma and chronic obstructive pulmonary disease. ACE indicates angiotensin-converting enzyme inhibitor; ARB, angiotensin receptor blocker; vertical line, March 2020, generally considered the beginning of the pandemic in the US.

Figure 1 shows rates of prescription fills for each drug group by month from January 2019 to December 2021. There is a pronounced increase in rates of statin and ACE and ARB fills in March 2020 (generally considered the beginning of the pandemic in the US), followed by a temporary downturn in May 2020. However, rates of 30-day fills increased in 2020 overall by 2.07% (95% CI, 2.01% to 2.12%) compared with 2019 (Table 2).

**Figure 2.**
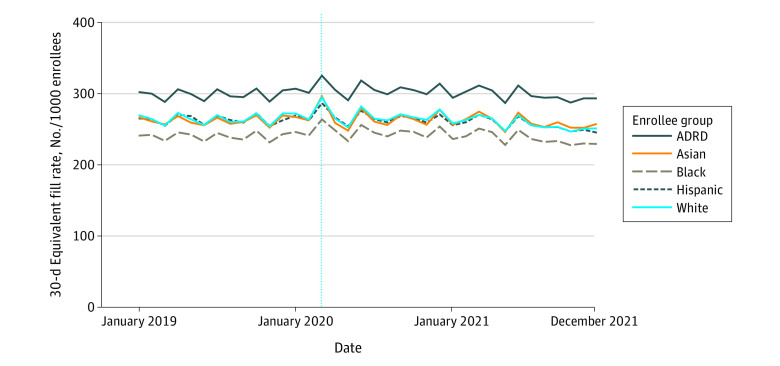
Overall Fill Rates for 5 Effective Treatments by Subgroup This figure displays monthly 30-day supply fill rates for an index of 5 common prescription drug groups for 6 groups of enrollees in Medicare Parts A, B, and D: Asian, Hispanic, non-Hispanic Black, and non-Hispanic White enrollees and those with an Alzheimer disease and related dementias (ADRD) diagnosis. All rates are per 1000 enrollees. The 5 drug groups are angiotensin-converting enzyme inhibitors and angiotensin receptor blockers, 3-hydroxy-3-methylglutaryl coenzyme A (HMG CoA) reductase inhibitors (statins), asthma and chronic obstructive pulmonary disease medications, antidepressants, and oral diabetes drugs. The vertical line indicates March 2020, generally considered the beginning of the pandemic in the US.

Figure 2 portrays mean fill rates for 5 drug groups for each study cohort. While mean rates differed, the temporal patterns of each group (Asian, Black, Hispanic, White, and ADRD populations) were similar. As shown in Table 2, there was a decline in the mean fill rate for all 5 groups (−2.61%, 95% CI, −2.67% to −2.56%) in 2021 compared with 2019, or approximately 470 000 enrollees. Within each drug group, the decline ranged from −5.19% (95% −CI, 5.25% to −5.14% for antidepressants to −1.22% (95% CI, −1.29% to −1.16%) for diabetes drugs.

Within each study cohort, the overall declines in fill rates were largest for Hispanic enrollees (−2.95%; 95% CI, −3.18% to −2.72%); the smallest declines were observed among Black enrollees (−1.42%; 95% CI, −1.64% to −1.20%), Asian enrollees (−1.05%; 95% CI, −1.36% to −0.77%), and enrollees diagnosed with dementia (−0.38%; 95% CI, −0.54% to −0.23%) (Table 2). Means across all drug groups mask cohort-specific heterogeneity across drug groups. For example, asthma and COPD medication fill rates declined from 2019 to 2021 by 8.21% (95% CI, −8.54% to −7.58%) for Asian enrollees but increased for Black enrollees (0.36%; 95% CI, 0.11% to 0.61%). Similarly, enrollees diagnosed with ADRD experienced a larger decrease in the receipt of ACEs and ARBs (−6.97%; 95% CI, −7.12% to −6.81%) but an increase in receipt of antidepressants (7.14%; 95% CI, 6.99% to 7.30%), which differed from the outcomes for other groups.

In 2019, the overall proportion of doses filled with a 90-day or greater supply per 100 fills was 75.91 fills (95% C.I., 75.89-75.93 fills), with corresponding rates by medication type ranging from 55.27 fills (95% CI, 55.25-55.29 fills) for asthma medications to 85.65 fills (95% CI, 85.63-85.66 fills) for ACEs and ARBs. Compared with 2019, the 2021 proportion of total fills per 100 fills dispensed as 90-day or greater supplies increased to 79.89 fills, an increase of 3.98 fills (95% CI, 3.94-4.03 fills). Larger changes per 100 fills were observed for Black enrollees (from 68.53 to 74.17 fills, an increase of 5.64 fills; 95% CI, 5.44-5.84 fills), Asian enrollees (73.40 to 78.11 fills, an increase of 4.72 fills; 95% CI, 4.46-4.98 fills), and Hispanic enrollees (72.27 to 76.94 fills, an increase of 4.67 fills; 95% CI, 4.46-4.88 fills). In contrast, enrollees diagnosed with ADRD experienced a smaller increase (0.55 fills; 95% CI, 0.40-0.70 fills) per 100 fills compared with the mean increase.

## Discussion

The early years of the COVID-19 pandemic provided important lessons about the provision of care and the risks to in-person services in future pandemics or other disruptive events.^1^ In this cohort study, we had hypothesized that disruption of in-person care during the pandemic would be associated with a decline in prescription receipt as a complement to in-person visits. We expected this to be apparent in the prescription receipt rate among older patients with chronic illness, particularly populations historically experiencing barriers to care and people with ADRD; care for these individuals is made more complex by dependence on informal caregivers, and they experienced disproportionate excess mortality during the pandemic.^11,18,19,20^ However, we found that overall receipt of drugs for chronic illness was relatively stable during the first 21 months of the pandemic. While there was evidence of larger declines in receipt of antidepressants for Hispanic Medicare enrollees and ACEs and ARBs for individuals diagnosed with ADRD, there were small overall mean differential changes in receipt by race, ethnicity, and ADRD diagnosis status in comparison with dramatic declines in hospitalizations and physician visits in the early phases of the pandemic.^1,2,3^ The increased use of larger drug fills (≥90-day supplies) may have been associated with smaller declines that otherwise may have been larger. The receipt of these larger supply fills increased more for Asian, Black, and Hispanic enrollees compared with the mean increase.

The surprising stability of prescription drug receipt during the pandemic may reflect well-established performance metrics that have long held Part D plans accountable for medication adherence among patients with chronic illness. Such performance metrics have promoted insurance plan features and beneficiary support aimed at maintaining medication receipt.^21^ To support adherence and increase drug-dispensing efficiency, pharmacies pivoted decades ago from a dependence on brick-and-mortar infrastructure to delivering medications to patient homes. When the pandemic began, pharmacies were already using home delivery and extended days-supply fills.^22^ Pharmacies and health plans were already complementing home delivery with telephone patient education, medication reconciliation, and adherence coaching for patients with high levels of need.^23^ While the Coronavirus Aid, Relief, and Economic Security (CARES) Act^24^ mandated 3-month supply dispensing for patients who requested it, our data revealed that this practice was commonplace before the mandate; 55% to 86% of 30-day supplies for medications considered in this study were received as 90-day or greater fills in 2019. A reduction in health plan denials of early refills may also have contributed to the observed overall fill rate stability.^4^

The relative increases in 90-day or greater supplies for prescription drugs among populations historically relatively underserved, however, were likely associated with only modest reductions in long-standing inequities in rates of effective chronic illness care receipt for these beneficiaries. Such inequities cannot be measured accurately from our data given that observed rates were relative to the entire population and not specific to individuals diagnosed with the disease; however, several studies^25,26,27^ document disparities in the use of effective care, such as statins and medications for diabetes.

These findings may suggest several lessons. Existing infrastructure and lack of dependence on face-to-face encounters were associated with resilience to the pandemic disruption of in-person care for prescription receipt. A similarly robust infrastructure to maintain clinician care provision in a future pandemic may mimic our current system of prescription delivery by fully enabling telehealth and telephone care as part of routine care-delivery models, reducing dependence on in-person visits and fostering care channel flexibility.^24,28^ While the topic is beyond the scope of this study, expanding telehealth and telephone care would require the same kinds of regulatory and reimbursement reforms already accomplished for prescription distribution. Whether similar robust infrastructure is feasible for additional services, such as cancer screening, is more speculative but may potentially be important.^29^ Additionally, the observed overall decrease of 2.61% in prescription fill rates suggests that approximately 470 000 of 18.11 million enrollees likely experienced a meaningful disruption of effective treatment. This and the differential decreases across populations and medication groups warrant exploration. Future research should examine factors and outcomes associated with this disruption.

Our findings parallel research demonstrating that long-term opioid therapy and treatments for addiction (such as buprenorphine) were relatively resilient to health care disruption during the pandemic.^30,31^ While restrictions around controlled substance prescribing and dispensing were relaxed modestly during the pandemic, the stability of access to these drugs among recipients before the pandemic, as with the medications we studied, suggests adaptation on the part of clinicians, pharmacies, and patients to maintain continuity despite dramatic disruption of health care delivery overall.^30,31^

### Limitations

Our analysis has several limitations. First, we implicitly assumed that the prevalence of diseases such as diabetes or hyperlipidemia (and hence demand for prescriptions) was relatively constant over the 3-year period. Evidence on diabetes^32^ and depression^33,34^ suggests that the pandemic was associated with a mixed outcome or a reduction in the prevalence of these conditions. Additionally, while post–COVID-19 condition was found to be associated with higher rates of cardiac disease,^35^ population-based rates of heart failure treatment, for example, have not increased.^36^ This and the lack of increase in fill rates during 2019 suggest that prevalence likely was not increasing substantially over this short period. Furthermore, we expect that the pandemic-related overall decrease in clinical encounters may have been associated with decreases in new diagnoses, providing opportunity to make and record such diagnoses. A decrease in the rate of new diagnoses would reduce the rate of new treatment starts, a component of our overall fill-rate measure.

Second, we measured prescription receipt at the population level and reported mean pandemic period changes relative to the prepandemic period. Our approach may miss substantial prescription receipt disruption among small subpopulations. Third, our study was limited to 5 common prescription drug groups generally used long term. While we believe that these drug groups are highly important and likely reflective of receipt of medications for chronic disease more generally, supply chain challenges or other factors may have been associated with more substantial changes in receipt of other drugs. Fourth, our sample was limited to enrollees in fee-for-service Medicare; findings may not be generalizable to other populations, such as individuals in Medicare Advantage or younger patients. For example, a 2023 study^37^ found increased adherence to asthma medication among US adults at midlife but a decline in prescriptions for children during the early pandemic.

Fifth, our study of diabetes medications did not include insulin because the days’ supply of insulin is difficult to interpret owing to variable (ie, sliding-scale) dosing. Sixth, our definition of ADRD was made based on a diagnosis at any point during 2019 to 2021, leading to attrition from the cohort because of mortality. The likely higher severity of disease in 2021 compared with 2019 would tend to bias our results toward a more rapid decline in prescription fills. Sixth, Medicare data were limited to 2019 to 2021, so we were not able to study trends prior to 2019 or prescription fills in 2022.

## Conclusions

This cohort study’s findings suggest that receipt of effective medications for chronic conditions was remarkably stable in the first 2 years of the COVID-19 pandemic compared with the prepandemic period and in stark contrast to other health services on which prescriptions are traditionally dependent (eg, face-to-face visits). This was likely associated with robust prescription dispensing and delivery infrastructure in the US and the uncoupling of prescription renewals from in-person visits. The stability may also reflect well-established performance metrics that have long held Part D plans accountable for medication adherence among patients with chronic illness and thus have promoted plan features and beneficiary support aimed at maintaining medication receipt. The increase in 90-day or greater supplies, especially in minority populations less likely to receive such supplies prior to the pandemic, may represent a small health care–delivery success for populations otherwise disproportionately negatively impacted by the pandemic. Our findings suggest that investment in remote care infrastructure and effective reimbursement schemes for other health care services that can be delivered safely without an in-person visit to a health care facility may be associated with reduced impact of future disruptive forces.

## Notes

Supplement 1. **eAppendix 1.** Identification of Beneficiaries Who Were Institutionalized for Study Exclusion **eAppendix 2.** Changes Over Time in Cohort Sample Sizes, 2019 to 2021 (Thousands) **eAppendix 3.** Identifying Beneficiaries With Alzheimer Disease and Related Dementias **eAppendix 4.** Medications Included in Each Drug Group of Interest **eAppendix 5.** CIs of Changes in Population-Based Rates of Prescription Fills
eReferences.

Supplement 2.
Data Sharing Statement
