# Supplementary material for: Receipt of Medications for Chronic Disease During the First 2 Years of the COVID-19 Pandemic Among Enrollees in Fee-for-Service Medicare
Source: JAMA Netw Open. 2023 May 17;6(5):e2313919. doi: 10.1001/jamanetworkopen.2023.13919 (PMC10193181; doi:10.1001/jamanetworkopen.2023.13919)
Supplement: Supplement 1. — eAppendix 1. Identification of Beneficiaries Who Were Institutionalized for Study Exclusion eAppendix 2. Changes Over Time in Cohort Sample Sizes, 2019 to 2021 (Thousands) eAppendix 3. Identifying Beneficiaries With Alzheimer Disease and Related Dementias eAppendix 4. Medications Included in Each Drug Group of Interest eAppendix 5. CIs of Changes in Population-Based Rates of Prescription Fills eReferences. [file jamanetwopen-e2313919-s001.pdf]

Supplemental Online Content

Morden NE, Zhou W, Obermeyer Z, Skinner J. Receipt of medications for chronic disease during the first 2 years of the COVID-19 pandemic among enrollees in fee-for-service Medicare. *JAMA Netw Open*. 2023;6(5):e2313919.
doi:10.1001/jamanetworkopen.2023.13919

eAppendix 1. Identification of Beneficiaries Who Were Institutionalized for Study Exclusion

eAppendix 2. Changes Over Time in Cohort Sample Sizes, 2019 to 2021 (Thousands)

eAppendix 3. Identifying Beneficiaries With Alzheimer Disease and Related Dementias

eAppendix 4. Medications Included in Each Drug Group of Interest

eAppendix 5. CIs of Changes in Population-Based Rates of Prescription Fills

eReferences.

This supplemental material has been provided by the authors to give readers additional information about their work.

© 2023 Morden NE et al. *JAMA Network Open.*

eAppendix 1. Identification of Beneficiaries Who Were Institutionalized for Study Exclusion

Nursing home residence was determined using place of service and Current Procedural Terminology codes from the Carrier (physician)and Outpatient files to identify enrollees with any nursing facility–related services in each month. If the beneficiary had 1 or more nursing facility–related place of service or Current Procedural Terminology codes in the relevant period, the beneficiary was classified as a nursing home resident.

As in past research, we employed the Yun et al. (2010)[1,2] algorithm to identify beneficiaries with any nursing facility service. During the study period, we identified beneficiaries as nursing home residents if the beneficiary had any one of the following:
(i) Any claim with POS codes (place of service codes: 31, 32,33) using Medicare Carrier File.
(ii) Any claim with CPT codes (99301, 99302, 99303, 99311, 99312, 99313, 99315, 99316, 99379, 99380, G0066) using Medicare Carrie and Outpatient Files
(iii) Any Skilled Nursing Facility (SNF) claim using Medicare SNF file.

© 2023 Morden NE et al. *JAMA Network Open.*

eAppendix 2. Changes Over Time in Cohort Sample Sizes, 2019 to 2021 (Thousands)

Month	All	Asian	Black	Hispanic	White	Other	ADRD
January 2019	18061	580	1152	940	14814	576	2476
2	18123	582	1154	941	14866	580	2471
3	18144	583	1153	942	14883	583	2442
4	18140	582	1148	937	14888	585	2405
5	18134	582	1141	931	14893	587	2376
6	18186	583	1141	932	14937	591	2367
7	18232	588	1142	936	14970	597	2329
8	18286	589	1143	937	15015	601	2308
9	18346	591	1145	939	15066	605	2292
10	18369	592	1145	941	15081	609	2251
11	18437	595	1151	947	15132	613	2242
12	18458	596	1153	950	15143	616	2208
January 2020	18050	585	1090	913	14851	611	2147
14	18088	585	1090	913	14886	614	2130
15	18123	586	1089	911	14921	616	2120
16	18220	589	1093	916	15001	621	2125
17	18237	590	1091	917	15015	624	2088
18	18244	591	1088	918	15020	626	2042
19	18282	594	1087	921	15049	631	2002
20	18301	595	1083	916	15073	634	1971
21	18328	596	1080	916	15099	637	1934
22	18334	596	1076	915	15107	639	1892
23	18383	599	1080	918	15143	642	1873
24	18377	600	1080	919	15133	644	1828
January 2021	17838	583	995	872	14753	634	1748
26	17843	584	992	870	14761	636	1709
27	17782	583	984	865	14715	636	1657
28	17772	581	978	858	14717	637	1621
29	17796	580	973	855	14748	639	1597
30	17796	581	970	854	14750	641	1559
31	17843	583	969	857	14787	645	1528
32	17877	584	968	859	14817	648	1500
33	17899	584	965	858	14841	650	1471
34	17898	584	961	857	14844	652	1429
35	17924	586	963	860	14861	655	1400
December 2021	17932	587	965	863	14861	656	1364
% Annual Change	-0.25	0.41	-5.55	-2.81	0.11	4.81	-15.39

© 2023 Morden NE et al. *JAMA Network Open.*

In this table we report the sample sizes (in thousands) by RTI race and ethnicity categories. (One limitation of the Medicare data is that it does not report ethnicity and race separately.) We did not report separate measures for "other" because it represents a combination of American Indian/Alaska Natives, "other," (typically more than one race/ethnicity) and unknown.

There was an annual decline in fee-for-service populations for Black enrollees (-5.55%) and Hispanic enrollees (-2.81%) enrollees. This is likely because of the rapid increases for Hispanic and Black Medicare enrollees in Medicare Advantage (Meyers et al., 2021). When fee-for-service Medicare enrollees sign up for Medicare Advantage, enrollment begins in January, which is consistent with the sudden drop in fee-for-service enrollment in January relative to December of the previous year. There could be an additional impact of higher mortality rates for Black and Hispanic enrollees during the COVID-19 pandemic (Gilstrap et al., 2022).

The largest decline is for ADRD patients (-15.39%) but this is the consequence of the way that the cohort was constructed. Because we were concerned that during the pandemic there would have been under-diagnosis of ADRD, we deemed anyone who had been diagnosed with ADRD during 2019-2021 to have ADRD for the entire period of analysis. Thus someone who was diagnosed with ADRD in 2020, and who died in 2021, would be in the "ADRD" cohort from January 2019 through their death in 2021. Because of the high rate of mortality among ADRD patients – almost 20% in 2019, prior to the pandemic, and nearly 25% in 2020 (Gilstrap et al., 2022), this means that the sample is expected, by construction, to experience attrition during the period of analysis.

© 2023 Morden NE et al. *JAMA Network Open.*

eAppendix 3. Identifying Beneficiaries With Alzheimer Disease and Related Dementias

Our definition of Alzheimer's Disease and Related Dementias (ADRD) was adopted from the Chronic Conditions Warehouse (CCW) algorithm[3]: at least one inpatient, HHA, HOP, or Carrier File claim with a ICD10 diagnosis code: F0150, F0151, F0280, F0281, F0390, F0391, F04, G138, F05, F061, F068, G300, G301, G308, G309, G311, G312, G3101, G3109, G94, R4181, R54 (any position on the claim). We limit the this designation to data from our study years only (2019-2021). In contrast, the CCW designation allows for measures over 3 years of look back.

The cohort of beneficiaries with Alzheimer's Disease and Related Dementias (ADRD) was defined as those with one or more diagnosis appearing on one or more claim 2019-2021. This inclusive approach was chosen to maximize ascertainment of beneficiaries with this condition for our monthly prescription fill measures. Dependence on monthly appearance of the diagnosis on a claim for the monthly cohorts would have missed many beneficiaries diagnosed with the condition.

© 2023 Morden NE et al. *JAMA Network Open.*

eAppendix 4. Medications Included in Each Drug Group of Interest

**Angiotensin Converting Enzymes and Angiotensin Receptor Blockers**
amlodipine besylate/benazepril HCl
azilsartan medoxomil
benazepril HCl
benazepril HCl/hydrochlorothiazide
candesartan cilexetil
captopril
captopril/hydrochlorothiazide
enalapril maleate
enalapril maleate/diltiazem malate
enalapril maleate/felodipine
enalapril maleate/hydrochlorothiazide
enalaprilat dihydrate
eprosartan mesylate
fosinopril sodium
fosinopril sodium/hydrochlorothiazide
irbesartan
lisinopril
lisinopril/hydrochlorothiazide
losartan potassium
moexipril HCl
moexipril HCl/hydrochlorothiazide
olmesartan medoxomil
perindopril arginine/amlodipine besylate
perindopril erbumine
quinapril HCl
quinapril HCl/hydrochlorothiazide
ramipril
telmisartan
trandolapril
trandolapril/verapamil HCl
valsartan
**Antidepressants**
amitriptyline HCl
amitriptyline HCl/chlordiazepoxide
amoxapine
benactyzine HCl

© 2023 Morden NE et al. *JAMA Network Open.*

brexanolone
bupropion HBr
bupropion HCl
bupropion HCl/dietary supplement combination no.15
bupropion HCl/dietary supplement combination no.16
citalopram hydrobromide
clomipramine HCl
desipramine HCl
desvenlafaxine
desvenlafaxine fumarate
desvenlafaxine succinate
doxepin HCl
duloxetine HCl
escitalopram oxalate
esketamine HCl
fluoxetine
fluoxetine HCl
fluoxetine HCl/dietary supplement no.17
fluoxetine HCl/dietary supplement no.8
fluvoxamine maleate
imipramine HCl
imipramine pamoate
isocarboxazid
levomilnacipran HCl
maprotiline HCl
milnacipran HCl
mirtazapine
nefazodone HCl
nortriptyline HCl
olanzapine/fluoxetine HCl
paroxetine HCl
paroxetine mesylate
perphenazine/amitriptyline HCl
phenelzine sulfate
protriptyline HCl
selegiline
sertraline HCl
tranylcypromine sulfate
trazodone HCl
trazodone HCl/dietary supplement no.8
trimipramine maleate

© 2023 Morden NE et al. *JAMA Network Open.*

venlafaxine HCl
vilazodone HCl
vortioxetine hydrobromide
**Asthma & Chronic Obstructive Pulmonary Disease**
aclidinium bromide
aclidinium bromide/formoterol fumarate
albuterol
albuterol sulfate
aminophylline
aminophylline in 0.9 % sodium chloride
aminophylline/ephedrine/potassium iodide/phenobarbital
arformoterol tartrate
beclomethasone dipropionate
benralizumab
bitolterol mesylate
budesonide
budesonide/formoterol fumarate
ciclesonide
clenbuterol HCl
cromolyn sodium
dexamethasone sodium phosphate
dupilumab
dyphylline
ephedrine HCl
ephedrine sulfate
ephedrine sulfate/guaifenesin
ephedrine/potassium iodide
epinephrine
epinephrine bitartrate
flunisolide
flunisolide/menthol
fluticasone furoate
fluticasone furoate/umeclidinium bromide/vilanterol trifenat
fluticasone furoate/vilanterol trifenatate
fluticasone propionate
fluticasone propionate, micronized
fluticasone propionate/salmeterol xinafoate
formoterol fumarate
formoterol fumarate dihydrate, micronized
glycopyrrolate
glycopyrrolate/formoterol fumarate

© 2023 Morden NE et al. *JAMA Network Open.*

glycopyrrolate/nebulizer accessories
glycopyrrolate/nebulizer and accessories
guaifenesin/dyphylline
guaifenesin/dyphylline/ephedrine/phenobarbital
guaifenesin/oxtriphylline
guaifenesin/theophylline
guaifenesin/theophylline anhydrous/pseudoephedrine
guaifenesin/theophylline/ephedrine
indacaterol maleate
indacaterol maleate/glycopyrrolate
ipratropium bromide
ipratropium bromide/albuterol sulfate
isoetharine HCl
isoetharine mesylate
isoproterenol HCl
isoproterenol sulfate
isoproterenol/calcium iodide
ketotifen fumarate
levalbuterol HCl
levalbuterol tartrate
mepolizumab
metaproterenol sulfate
mometasone furoate
mometasone furoate/formoterol fumarate
montelukast sodium
nedocromil sodium
olodaterol HCl
omalizumab
oxtriphylline
pirbuterol acetate
racepinephrine HCl
reslizumab
revefenacin
roflumilast
salmeterol xinafoate
terbutaline sulfate
theophylline anhydrous
theophylline in dextrose 5 % in water
theophylline/caffeine/AA no.13/cinnamon/herbal 135
theophylline/dietary supplement,misc.comb.no.9
theophylline/ephedrine HCl/phenobarbital

© 2023 Morden NE et al. *JAMA Network Open.*

theophylline/ephedrine/hydroxyzine
theophylline/ephedrine/potassium iodide/phenobarbital
theophylline/potassium iodide
tiotropium bromide
tiotropium bromide/olodaterol HCl
tranilast
triamcinolone acetonide
umeclidinium bromide
umeclidinium bromide/vilanterol trifenatate
zafirlukast
zileuton
**Diabetes Medications**
acarbose
acetohexamide
alogliptin benzoate
alogliptin benzoate/metformin HCl
alogliptin benzoate/pioglitazone HCl
canagliflozin
canagliflozin/metformin HCl
chlorpropamide
dapagliflozin propanediol
dapagliflozin propanediol/metformin HCl
dapagliflozin propanediol/saxagliptin HCl
empagliflozin
empagliflozin/linagliptin
empagliflozin/linagliptin/metformin HCl
empagliflozin/metformin HCl
ertugliflozin pidolate
ertugliflozin pidolate/metformin HCl
ertugliflozin pidolate/sitagliptin phosphate
glimepiride
glipizide
glipizide/metformin HCl
glyburide
glyburide,micronized
glyburide/metformin HCl
linagliptin
linagliptin/metformin HCl
miglitol
nateglinide
pioglitazone HCl

© 2023 Morden NE et al. *JAMA Network Open.*

pioglitazone HCl/glimepiride
pioglitazone HCl/metformin HCl
repaglinide
repaglinide/metformin HCl
rosiglitazone maleate
rosiglitazone maleate/glimepiride
rosiglitazone maleate/metformin HCl
saxagliptin HCl
saxagliptin HCl/metformin HCl
sitagliptin phosphate
sitagliptin phosphate/metformin HCl
sitagliptin phosphate/simvastatin
tolazamide
tolbutamide
troglitazone
**Statins (HMG CoA Reductase Inhibitors)**
amlodipine besylate/atorvastatin calcium
atorvastatin calcium
cerivastatin sodium
fluvastatin sodium
lovastatin
niacin/lovastatin
niacin/simvastatin
pitavastatin calcium
pitavastatin magnesium
pravastatin sodium
rosuvastatin calcium
simvastatin

Drug groups were created using the First Data Bank drug grouping software to identify National Drug Code (NDC) lists for products in the drug groups of interest. (First Databank, 2022).

© 2023 Morden NE et al. *JAMA Network Open.*

eAppendix 5. CIs of Changes in Population-Based Rates of Prescription Fills

In this section, we show how we calculate confidence intervals for our analysis of proportional declines. Let y be the estimated average fill rate for (say) 2021 and x the corresponding estimated fill rate in 2019. The estimated proportional change, d, is therefore

A.1 $d = (y - x) / x$

Assuming independence for the sample rates in the two years, the variance of the estimate of d can be approximated using the delta method by

A.2 $Var(d) \cong Var(x)[E(y) / (E(x)^2] + Var(y) / E(x)$

Confidence intervals can then be calculated in the usual way (see the Supplemental documentation for Gilstrap et al., 2022). We adopt the conservative approach of using as the sample size the average enrollment for the entire year despite the presence of multiple fills within each year. Variances are calculated using the exact binomial approach.

For the index (or average) measures across the 5 treatment groups, we account for the positive covariance of the fills by month; that is, in months where diabetes drugs rise or fall, there is empirical evidence of a greater likelihood of (e.g.) anti-depressants rising and falling as well. (This has the effect again of making the confidence intervals wider than if the 5 fill rates were independent.) Defining $\overline{z}$ as the average across the five drug classes in a given year:

A.3 $$\overline{z} = \sum_{k=1}^{5} \frac{z_k}{5}$$

The variance of $\overline{z}$ is therefore written:

A.4 $$Var(\overline{z}) = \left[\sum_{k=1}^{5} \left(Var(z_k) + \sum_{j \neq k}^{5} Cov(z_j, z_k) \right) \right] / 5$$

The monthly data on each of the five measures during the pre-pandemic period exhibits a high degree of correlation, on the order of 0.8 to 0.9. This means that assuming that the index consists of 5 independent random variables will lead to a confidence interval that is too tight. For this reason, we assume the extreme case that each of these 5 components are perfectly correlated (e.g., the correlation coefficient is equal to one), it is straightforward to show that the variance of the index is written as:

A.5 $$Var(\overline{z}) = \left[\sum_{k=1}^{5} \left(se(z_k) / 5 \right) \right]^2$$

Where se denotes standard error. That is, the standard error of $\overline{z}$ is simply the average of the standard errors of each of its components. This leads to a conservative estimate of the confidence intervals for the index; as the correlation coefficient shifts from 1 to zero, the confidence intervals become successively tighter.

© 2023 Morden NE et al. *JAMA Network Open.*

In calculating confidence intervals for the changes in the fraction of fills that were 90+ days, we calculated the standard errors of the difference in rates assuming independence.

© 2023 Morden NE et al. *JAMA Network Open.*

eReferences

Birkmeyer, J.D., Barnato, A., Birkmeyer, N., Bessler, R. and Skinner, J., 2020. The impact of the COVID-19 pandemic on hospital admissions in the United States: study examines trends in US hospital admissions during the COVID-19 pandemic. Health Affairs, 39(11), pp.2010-2017.

First Databank. FDB MedKnowledge. Accessed December 9, 2022. https://www.fdbhealth.com/solutions/medknowledge-drug-database

Gilstrap, L., Zhou, W., Alsan, M., Nanda, A. and Skinner, J.S., 2022. Trends in mortality rates among Medicare enrollees with Alzheimer disease and related dementias before and during the early phase of the COVID-19 pandemic. JAMA neurology, 79(4), pp.342-348.

Meyers, D.J., Mor, V., Rahman, M. and Trivedi, A.N., 2021. Growth In Medicare Advantage Greatest Among Black And Hispanic Enrollees: Study examines the extent to which growth in Medicare Advantage is being driven by increased participation of racial/ethnic minorities and other traditionally marginalized groups. Health Affairs, 40(6), pp.945-950.

(ResDAC) RDAC. Data Documentation, Part D Event (PDE) File. Accessed November 11, 2022. https://resdac.org/cms-data/files/pde

Yun, H., Kilgore, M.L., Curtis, J.R., Delzell, E., Gary, L.C., Saag, K.G., Morrisey, M.A., Becker, D., Matthews, R., Smith, W. and Locher, J.L., 2010. Identifying types of nursing facility stays using Medicare claims data: an algorithm and validation. Health Services and Outcomes Research Methodology, 10, pp.100-110.

© 2023 Morden NE et al. *JAMA Network Open.*
