# Supplementary material for: Receipt of Medications for Chronic Disease During the First 2 Years of the COVID-19 Pandemic Among Enrollees in Fee-for-Service Medicare
Source: JAMA Netw Open. 2023 May 17;6(5):e2313919. doi: 10.1001/jamanetworkopen.2023.13919 (PMC10193181; doi:10.1001/jamanetworkopen.2023.13919)
Supplement: Supplement 2. — Data Sharing Statement [file jamanetwopen-e2313919-s002.pdf]

Data Sharing Statement

Morden. Receipt of Medications for Chronic Disease During the First 2 Years of the COVID-19 Pandemic Among Enrollees in Fee-for-Service Medicare. *JAMA Netw Open*. Published online May 17, 2023. doi:10.1001/jamanetworkopen.2023.13919

Data
Data available: We have posted (a) SAS programs to create the month x cohort x drug classification matrix for rates of prescribing from the CMS Medicare claims data, (b) Stata files to create Table 2 and Figures 1 and 2, and (c) Excel spreadsheets with the intermediate month x cohort x drug classification measures on the following website:

https://www.nber.org/chronic-medication-receipt-during-first-two-years-covid-19-pandemic-programs-and-data

We cannot provide the individual level Medicare claims data.

Additional Information
Explanation for why individual Medicare claims data not available: Our DUA with CMS does not permit data sharing.

© 2023 Morden NE et al. *JAMA Network Open*.
